# Observational Study of Sublingual Microcirculation in Patients With Chronic Cardiovascular Disease

**DOI:** 10.1111/micc.70032

**Published:** 2025-10-13

**Authors:** Jacob Niculcea, James W. Schurr, Fatima M. Talebi, Joyce W. Wald, John C. Greenwood

**Affiliations:** ^1^ Division of Cardiovascular Medicine, Perelman School of Medicine at the University of Pennsylvania Hospital of the University of Pennsylvania Philadelphia Pennsylvania USA; ^2^ Touro College of Osteopathic Medicine New York New York USA; ^3^ Department of Emergency Medicine, Perelman School of Medicine at the University of Pennsylvania Hospital of the University of Pennsylvania Philadelphia Pennsylvania USA; ^4^ Department of Anesthesiology and Critical Care, Perelman School of Medicine at the University of Pennsylvania Hospital of the University of Pennsylvania Philadelphia Pennsylvania USA

## Abstract

**Background:**

Sublingual video microscopy is increasingly used to study the microcirculation in acute illness. However, the sublingual microcirculation in patients with chronic cardiovascular diseases (CVD) is understudied. Our objective was to quantify sublingual microcirculatory parameters in a large cohort of patients with CVD.

**Methods:**

One hundred and thirteen patients with CVD were enrolled. Incident dark‐field handheld video microscopy (IDF‐HVM) was used to quantify microvascular flow index (MFI), microvascular heterogeneity index (MHI), proportion of perfused vessels (PPV), perfused vessel density (PVD), and total vessel density (TVD). Data were stratified by age quartiles (20–39, 40–59, 60–79, 80+), cardiovascular comorbidities (hypertension, diabetes, chronic kidney disease, coronary artery disease, heart failure), and systemic hemodynamics (mean arterial pressure and pulse pressure).

**Results:**

In our 113 patient cohort, overall MFI = 2.86 ± 0.20; MHI = 0.15 ± 0.20; PPV = 94.3% ± 4.9%; PVD = 23.1 ± 4.7 mm/mm^2^; and TVD = 24.5 ± 4.8 mm/mm^2^. Diabetic patients had lower mean MHI (0.10 vs. 0.17; *p* = 0.046) compared to those without diabetes. There was no difference in sublingual parameters attributable to other CVDs, age, or hemodynamics.

**Conclusion:**

In patients with stable cardiovascular disease, sublingual microvascular parameters are similar across age, blood pressure, and comorbidity cohorts, with the exception of decreased MHI in diabetic patients.

**Abbreviations**
CAD: coronary artery disease
CKD: chronic kidney disease
DM: diabetes mellitus
HFrEF: heart failure with reduced ejection fraction
HTN: hypertension
HVM: handheld video microscopy
IDF‐HVM: incident darkfield handheld video microscopy
MAP: mean arterial pressure
MFI: microvascular flow index
MHI: microvascular heterogeneity index
PPV: proportion of perfused vessels
PVD: perfused vessel density
TVD: total vessel density

## 1 Introduction

The microcirculation is a complex network of small diameter blood vessels less than 100 μm in diameter including arterioles, capillaries, and venules [1]. Healthy microcirculatory blood flow is critical for oxygen delivery, cellular metabolism, immune cell delivery, and tissue fluid balance. Progressive endothelial dysfunction, decreased vascular reactivity, rarefaction, hyalinization, vessel thickening, and loss of glycocalyx integrity due to chronic cardiovascular disease may result in functional changes over time [2, 3, 4, 5].

Sublingual handheld video microscopy (HVM) is a non‐invasive method for quantifying microcirculatory blood flow in humans [6]. Handheld video microscopy makes possible the rapid, in vivo analysis of microvessel density as well as semi‐quantitative analysis of microvascular blood flow and flow heterogeneity [7]. The rapid and non‐invasive nature of HVM is particularly useful for studying acute circulatory shock in the critical care setting, where microvascular dysfunction is integral to understanding the pathogenesis of shock [8]. Sublingual microcirculation measurements have been shown to correlate with other end organ capillary beds, most notably, the intestinal microcirculation [9]. For this reason, sublingual imaging is an accessible surrogate for understanding the microcirculatory density and blood flow of internal organs which are often injured during periods of shock [10]. Sublingual microcirculation parameters such as decreased perfused vessel density and increased flow heterogeneity have been associated with worse patient outcomes and disease severity in acute illness [11, 12, 13, 14, 15].

Currently, less is known about sublingual microcirculation in patients with stable, chronic cardiovascular diseases (CVD). Chronic cardiovascular disease and aging are known to cause changes to microvascular structures and blood flow, but this population is not the focus of most research using HVM [16]. It is currently unknown whether HVM can detect microcirculation abnormalities present with chronic CVD.

A better understanding of sublingual microcirculation in patients with cardiovascular disease (CVD) has important implications for the study of acute illness. Currently, no standardized reference ranges exist for sublingual microcirculatory parameters such as vessel density, blood flow, or flow heterogeneity [17]. This lack of normative data limits the interpretability of microcirculatory assessments in acutely ill patients, as comparisons to stable, comorbidity‐matched controls are not readily available. In this study, we characterize sublingual microcirculatory hemodynamics in stable, ambulatory patients with CVD using handheld vital microscopy (HVM), reporting standard measurements of vessel density, flow, and heterogeneity in accordance with established consensus guidelines [18].

## 2 Methods

### 2.1 Study Design

This study is a prospective, observational study that enrolled patients with known CVD as part of the larger MicroRESUS study at the University of Pennsylvania [19]. The study was approved by the local Institutional Review Board (#829765).

### 2.2 Patient Population

Adult patients (≥ 18 years old) were approached in an outpatient setting during a routine clinic visit or as part of an evaluation for possible cardiovascular surgery for informed consent. Efforts were maintained to include a diverse patient sample that considered age, race, ethnicity, and gender. Subjects were excluded if they had abnormal vital signs (heart rate > 100 beats per minute, systolic blood pressure ≤ 90 mmHg), evidence of hypoxemia (SpO_2_ < 92% on room air), acute illness, evidence of new end‐organ injury on outpatient laboratory testing, congenital heart disease, or were unable to tolerate sublingual microcirculatory flow imaging with the handheld video microscope.

### 2.3 Cardiovascular Disease and Co‐Morbid Conditions

We defined CVD as the presence of at least one of the following diagnoses: hypertension (HTN), hyperlipidemia (HLD), coronary artery disease (CAD), ischemic or non‐ischemic cardiomyopathy (NICM), heart failure (HF), valvular disorders, and peripheral arterial disease (PAD) [20].

### 2.4 Microcirculation Measurement

Sublingual microcirculation imaging was performed using incident darkfield (IDF) handheld video microscopy (IDF‐HVM; CytoCam, Braedius Medical BV, the Netherlands) immediately after enrollment. Imaging was performed by the Principal Investigator (J.C.G.) and a trained research assistant (F.M.T.) while subjects were resting in a semi‐recumbent position with the head of the bed at 45°. A series of successive video clips (at least 3 video sequences with 120 frames) were captured in at least 3 distinct areas of the sublingual triangle to account for anatomic and flow heterogeneity [21]. Attention was made to quality factors during subject imaging to avoid pressure artifact, excess saliva, and proper location in accordance with the accepted consensus standards for microcirculation imaging [18, 22]. Each clip was deidentified, coded, and analyzed after enrollment was complete.

### 2.5 Analysis of IDF Video Microscopy

**FIGURE 1:**
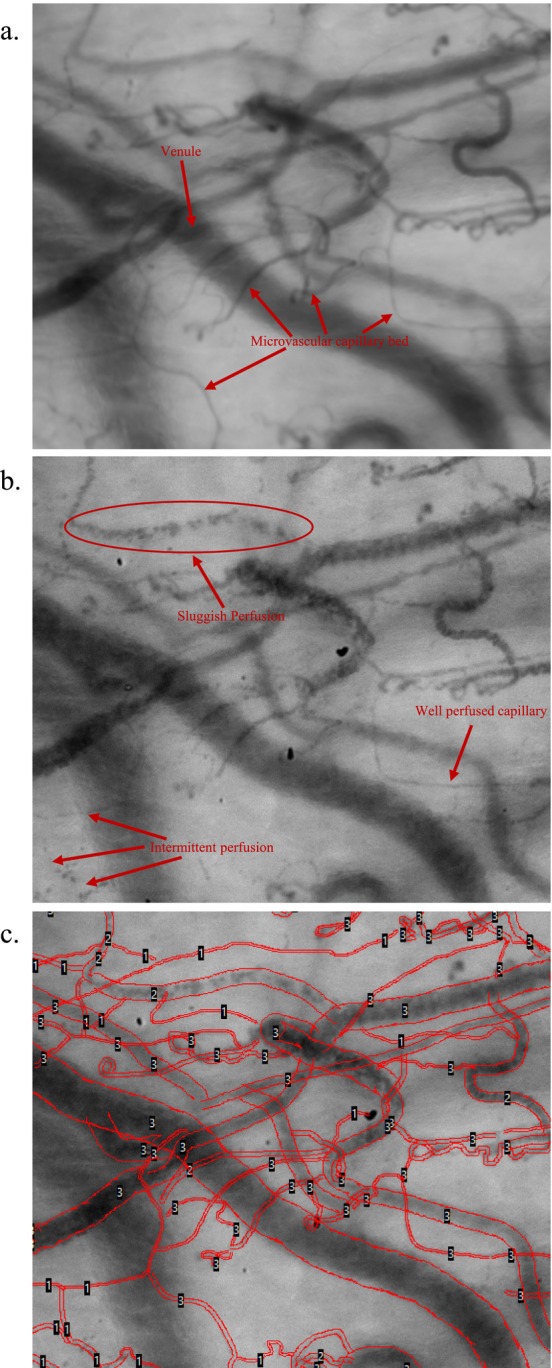
Screenshots from microcirculatory imaging analysis (a) Composite image of 80 frames of sublingual microvascular video, allowing clear visualization of capillaries, arterioles, and venules. (b) Single frame/image of the sublingual microvascular bed, noting vessels that are well, sluggishly, and intermittently perfused. (c) Hand tracing of the microcirculatory bed with scoring of flow in each vessel based on the scale described in Table 2.

Prior to analysis, video quality was assessed using the 6‐factor Massey quality score [22]. Only videos with Massey scores of < 10 were included. Manual analysis was performed with Automated Vascular Analysis (AVA 3.2; Microvision Medical BV, the Netherlands) software by two investigators (JCG, FMT) who were blinded to the subject's clinical data (Figure 1). Only vessels ≤ 20 μm in diameter were included in the analysis. Microvascular flow index (MFI), microcirculatory heterogeneity index (MHI), total vessel length (TVL), total vessel density (TVD), proportion of perfused vessels (PPV), and perfused vessel density (PVD) were calculated (Table 1). Vessels were considered perfused if their flow score was graded as sluggish or continuous flow. Interobserver variation was evaluated in 10% of the subject videos to ensure minimal scoring heterogeneity between investigators.

**TABLE 1:** Description of basic sublingual microscopy parameters.

Microcirculatory parameter	Background	Relevant calculation	Units
MFI (microvascular flow index)	Video clips are divided into quadrants and aggregate flow in each quadrant is graded manually by the user on a nominal scale (0 = no flow, 1 = intermittent flow, 2 = sluggish flow, 3 = continuous flow). MFI is the average score across 4 quadrants.	n/a	Arbitrary/Unitless
MHI (Microvascular heterogeneity index)	Estimate of the heterogeneity of blood flow among different areas within a capillary bed.	MHI = (MFI_Max_−MFI_Min_)/MFI_Mean_	Arbitrary/Unitless
TVL (Total vessel length)	Vessels are traced by hand (gold standard) or by semi‐automated software (see Figure 1C). Vessel length in mm is then estimated via spatial calibration.	n/a	mm
TVD (Total vessel density)	TVL is divided by the total area of the imaged capillary bed to estimate the density of vessels in the capillary bed.	TVD = TVL/Total area of the imaged capillary bed	1/mm
PPV (Proportion of perfused vessels)	Blood flow in each traced vessel is graded manually on the same nominal scale as MFI. Only vessels graded 2 or 3 are considered perfused.	PPV = (# of vessels graded 2 or 3/Total # of Vessels) ×100	%
PVD (Perfused vessel density)	Provides an estimate of functional vessel density (i.e., only accounting for vessels graded 2 or 3)	PVD = (Total length of vessels graded 2 or 3)/total area of imaged capillary bed	1/mm

### 2.6 Statistical Analysis

Data normality was assessed with Shapiro–Wilk testing. Continuous variables characterizing demographic data and microcirculation data were reported as means with standard deviations when normally distributed or medians with interquartile ranges when not normally distributed. Comparison of continuous variables according to age groups was done with the one‐way ANOVA test. Categorical variables, including patient comorbidities, are represented as frequencies with proportions and compared using chi‐squared or Fisher exact test. Correlation between continuous variables was performed using simple linear regression reporting Pearson coefficients. All statistical analyses were performed with R (R Foundation for Statistical Computing; Version 4.3.3.).

## 3 Results

### 3.1 Patient Characteristics and Aggregated Microcirculatory Parameters

The study population consisted of 113 adult patients (mean age 63 ± 13 years) recruited during clinic visits to evaluate patients for elective cardiovascular surgery (Table 2). 67% of patients were male; 33% were female. 62% of patients had chronic hypertension; 34% of patients had diabetes mellitus; 20% of patients had chronic kidney disease; 43% had coronary artery disease; and 16% had heart failure with reduced ejection fraction (HFrEF). The average body mass index was 28 ± 6 kg/m^2^. Mean estimated glomerular filtration rate (eGFR) was 41 ± 33 mL/min/1.73 m^2^.

**TABLE 2:** Baseline study population, comorbid conditions, and labs.

Baseline: Characteristics	*N* = 133; Mean ± SD; (%)
Age, years	63 ± 13
Sex	
Female	37 (33%)
Male	76 (67%)
Height (cm)	173 ± 11
BMI (kg/m^2^)	28 ± 6
Race	
American Indian	0 (0%)
Asian	1 (0.9%)
Pacific Islander	0 (0%)
Black/African American	11 (9.7%)
White	100 (88%)
More than one race	1 (0.9%)
Ethnicity	
Hispanic/Latino	5 (4.5%)
Not Hispanic/Latino	107 (96%)
Comorbidities	
Hypertension	70 (62%)
Diabetes	38 (34%)
COPD	9 (8.0%)
CKD	23 (20%)
EGFR (mL/min/1.73 m^2^)	76 ± 27
CAD	49 (43%)
HFrEF	18 (16%)
Labs	
Hemoglobin (g/dL)	12.97 ± 2.25
Hematocrit (%)	38.1 ± 6.1
WBC (/μL)	7.10 ± 2.86
Platelets (/μL)	220 ± 79
INR	1.08 ± 0.13
aPTT (s)	39 ± 24
BUN (mg/dL)	20 ± 9
Cr (mg/dL)	1.34 ± 1.80
AST (U/L)	27 ± 17
ALT (U/L)	29 ± 24
Bilirubin (mg/dL)	0.74 ± 0.43
Lactate (mmol/L)	0.86 ± 0.39

### 3.2 Microcirculation Imaging Analysis

A total of 364 imaging sequences with 43 680 individual video frames were included in the study. The total median [interquartile range] Massey quality score for all videos was 0 [0–1].

Across all patients in our study, microcirculatory data is reported in Table 3. This data reflects mean sublingual microvessel density (TVD), flow (MFI, PPV, PVD), and flow heterogeneity (MHI). Subsequent subgroup analysis stratifying by age, specific comorbidities, and macrocirculatory hemodynamics was performed.

**TABLE 3:** Mean microcirculatory parameters for all patients.

Microcirculation parameters	
Characteristic	*N* = 113^a^
MFI	2.86 ± 0.20, 2.94
MHI	0.15 ± 0.20, 0.09
PPV	94.3 ± 4.9, 95.7
PVD	23.1 ± 4.7, 22.6
TVD	24.5 ± 4.8, 23.6

^a^ Mean ± SD, Median.

### 3.3 Microcirculatory Parameters Stratified by Age and Comorbidities

**FIGURE 2:**
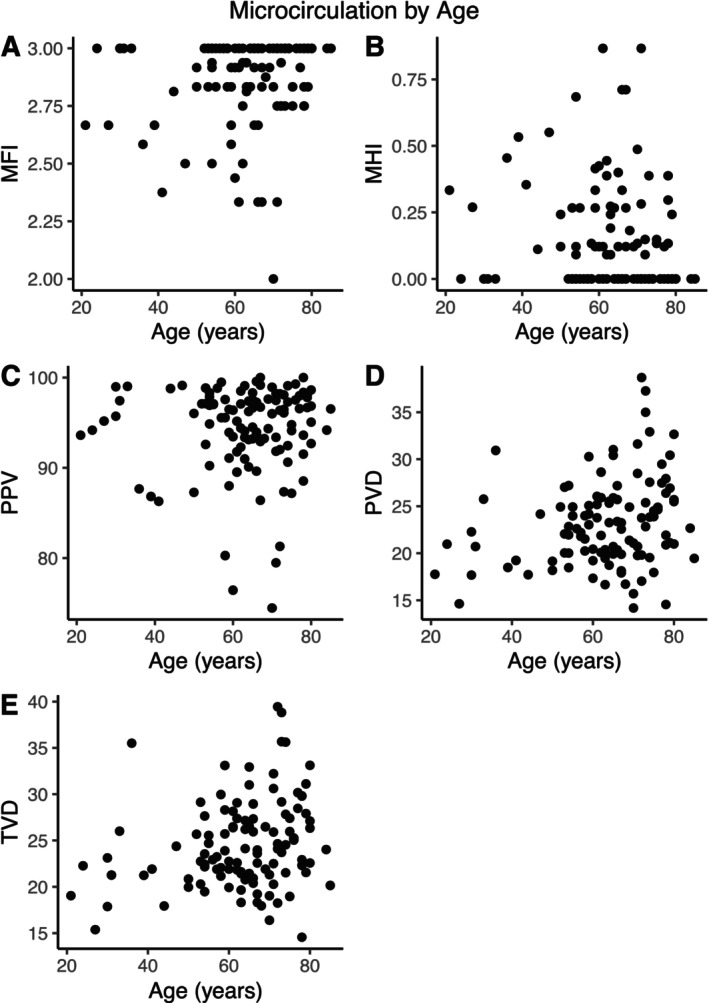
Microcirculatory parameters stratified by patient age. (A) MFI by age. (B) MHI by age. (C) PPV by age. (D) PVD by age. (E) TVD by age.

**FIGURE 3:**
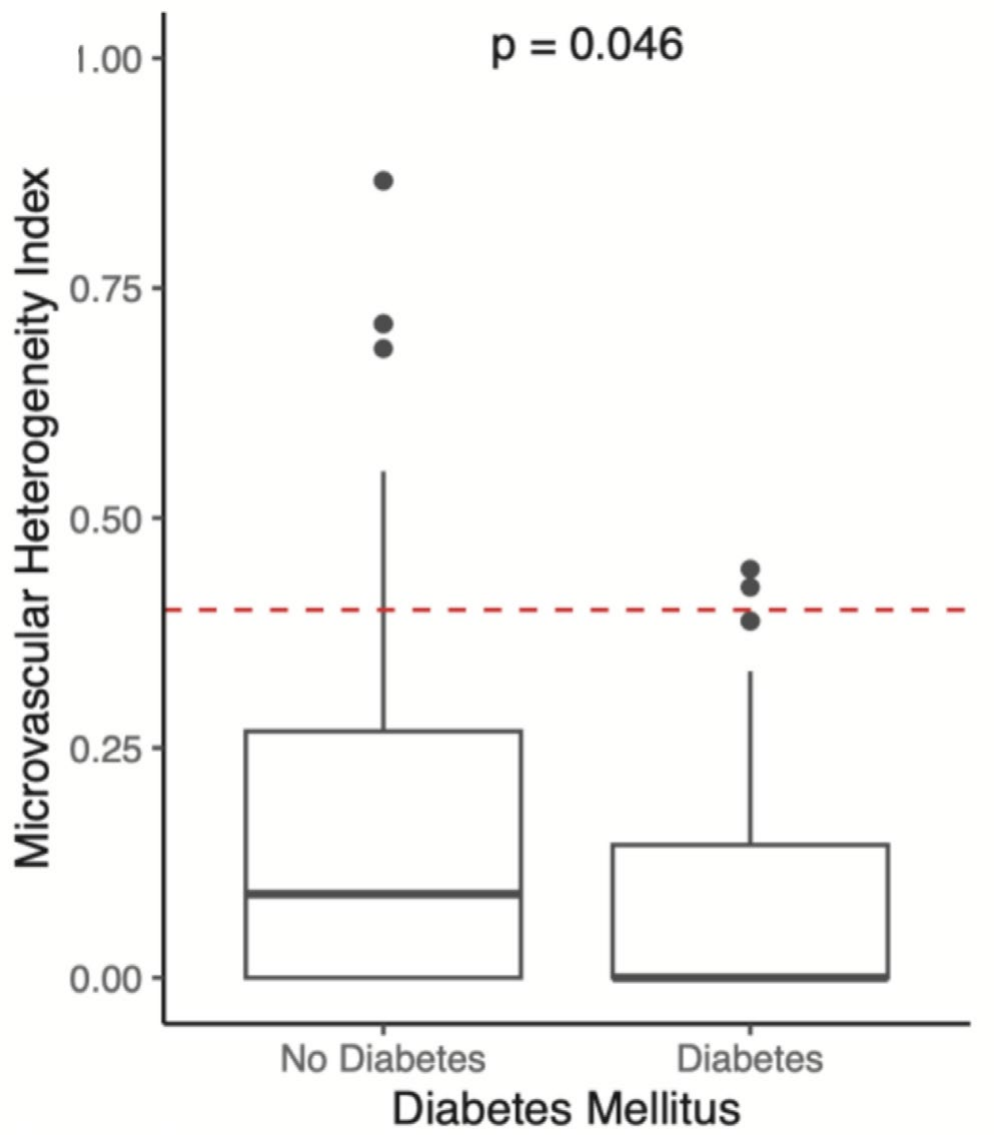
Effects of DM on MHI.

There were no differences between microcirculatory perfusion parameters when stratified by age group (20–39, 40–59, 60–79, 80+; Table 4, Figure 2). Microcirculation parameters were generally similar when compared across comorbid conditions, including HTN, DM, CAD, HFrEF, CKD by stage (Tables 5 and S1, Figures S1–S5). Patients with diabetes had lower microcirculation flow heterogeneity (MHI 0.10 vs. 0.17; *p* = 0.046) compared to non‐diabetic patients (Table 5, Figure 3). All other microcirculatory parameters showed no changes with respect to comorbid conditions.

**TABLE 4:** Microcirculation parameters stratified by age group.

Microcirculation parameters by age group					
Characteristic	20–39 years	40–59 years	60–79 years	80+ years	*p* ^b^
	*N* = 10^a^	*N* = 29^a^	*N* = 72^a^	*N* = 2^a^	
Age, years	31 ± 6, 31	55 ± 5, 55	70 ± 6, 71	85 ± 1, 85	< 0.001
MFI	2.83 ± 0.19, 2.83	2.85 ± 0.19, 2.92	2.87 ± 0.20, 3.00	3.00 ± 0.00, 3.00	0.7
MHI	0.21 ± 0.24, 0.13	0.16 ± 0.19, 0.12	0.14 ± 0.21, 0.00	0.00 ± 0.00, 0.00	0.5
PPV	93.5 ± 4.8, 94.7	94.1 ± 5.7, 96.4	94.4 ± 4.7, 95.6	95.4 ± 1.7, 95.4	> 0.9
PVD	20.8 ± 4.7, 19.6	22.2 ± 3.1, 22.1	23.8 ± 5.1, 23.6	21.1 ± 2.3, 21.1	0.13
TVD	22.3 ± 5.5, 21.3	23.6 ± 3.4, 22.7	25.2 ± 5.1, 24.9	22.1 ± 2.7, 22.1	0.2

^a^ Mean ± SD, Median.

^b^ Signifcance level: P < 0.05.

**TABLE 5:** Mean microcirculatory parameters for comorbidity subpopulations (HTN, DM, CAD, HFrEF, CKD).

Microcirculation parameters by comorbidity															
Characteristic	−HTN	+HTN	*p*	−DM	+DM	*p*	−CAD	+CAD	*p*	−HFrEF	+HFrEF	*p*	−CKD	+CKD	*p*
N	43	70	n/a	76	38	n/a	64	49	n/a	95	18	n/a	90	23	n/a
Age, years	59 ± 16, 60	66 ± 11, 66	0.017	62 ± 14, 65	64 ± 12, 66	0.4	60 ± 15, 63	67 ± 10, 67	0.005	62 ± 14, 65	67 ± 11, 70	0.2	61 ± 14, 64	69 ± 11, 71	0.008
MFI	2.88 ± 0.17, 3.00	2.85 ± 0.21, 2.93	0.4	2.85 ± 0.22, 2.94	2.90 ± 0.14, 3.00	0.11	2.84 ± 0.21, 2.92	2.89 ± 0.18, 3.00	0.2	2.87 ± 0.20, 3.00	2.84 ± 0.21, 2.93	0.6	2.86 ± 0.21, 2.97	2.89 ± 0.16, 2.94	0.4
MHI	0.12 ± 0.17, 0.00	0.17 ± 0.22, 0.11	0.2	0.17 ± 0.23, 0.09	0.10 ± 0.14, 0.00	0.046*	0.17 ± 0.21, 0.12	0.13 ± 0.20, 0.00	0.3	0.15 ± 0.21, 0.00	0.16 ± 0.19, 0.11	0.7	0.15 ± 0.21, 0.05	0.13 ± 0.20, 0.09	0.6
PPV	94.7 ± 4.8, 96.4	94.0 ± 5.0, 95.0	0.5	94.1 ± 4.7, 95.2	94.7 ± 5.4, 96.7	0.5	94.4 ± 5.4, 96.1	94.1 ± 4.3, 95.5	0.8	94.5 ± 4.6, 95.7	93.1 ± 6.3, 95.7	0.4	94.4 ± 4.8, 95.6	93.9 ± 5.5, 96.0	0.7
PVD	22.4 ± 4.3, 22.0	23.5 ± 4.8, 23.3	0.2	23.0 ± 5.0, 22.1	23.1 ± 4.1, 22.9	> 0.9	23.0 ± 4.8, 22.7	23.2 ± 4.6, 22.0	0.8	23.2 ± 4.9, 22.2	22.5 ± 3.2, 23.2	0.5	23.3 ± 4.4, 22.8	22.2 ± 5.5, 20.7	0.4
TVD	23.7 ± 4.6, 22.6	25.0 ± 4.9, 24.5	0.2	24.5 ± 5.1, 23.1	24.4 ± 4.2, 24.0	> 0.9	24.4 ± 4.8, 23.6	24.6 ± 4.8, 23.6	0.8	24.5 ± 5.1, 23.1	24.1 ± 2.8, 24.1	0.6	24.7 ± 4.5, 23.7	23.7 ± 5.8, 21.9	0.4

*Note:* Mean ± SD, Median. Significance level: P < 0.05.

### 3.4 Microcirculatory Parameters Stratified by Macrocirculatory Hemodynamics

We also investigated the effects of macrocirculatory hemodynamics [mean arterial blood pressure (MAP) and pulse pressure] on microcirculation. Average MAP was 98.9 ± 59.1 mmHg and mean pulse pressure was 60.4 ± 20.7 mmHg. Neither MAP nor pulse pressure had a statistically significant effect on microcirculatory parameters (Tables 6 and 7, Figure S6,S7). Notably, mean PVD and TVD trended toward statistical significance when stratified by MAP quartiles (*p =* 0.052, 0.050, and 0.058, respectively).

**TABLE 6:** Microcirculation parameters stratified by macrocirculatory hemodynamics (MAP).

Microcirculation parameters by mean arterial blood pressure (Quartiles)					
Characteristic	Lowest	Low‐Middle	High‐Middle	Highest (104+)	*p* ^b^
	(62–81 mmHg)	(82–94 mmHg)	(95–104 mmHg)		
	*N* = 29^a^	*N* = 26^a^	*N* = 28^a^	*N* = 29^a^	
Age, years	66 ± 12, 65	57 ± 19, 61	64 ± 13, 67	65 ± 9, 66	0.077
MFI	2.89 ± 0.16, 2.92	2.87 ± 0.22, 3.00	2.86 ± 0.19, 2.97	2.86 ± 0.22, 2.94	> 0.9
MHI	0.15 ± 0.21, 0.11	0.12 ± 0.16, 0.00	0.15 ± 0.20, 0.05	0.16 ± 0.24, 0.09	> 0.9
PPV	94.3 ± 3.6, 94.5	94.5 ± 5.8, 96.4	94.3 ± 3.8, 93.6	94.5 ± 5.3, 96.6	> 0.9
PVD	23.6 ± 5.4, 22.8	21.3 ± 4.0, 20.7	24.7 ± 5.1, 24.0	22.7 ± 3.4, 22.0	0.052
TVD	25.0 ± 5.3, 24.0	22.6 ± 4.5, 22.1	26.1 ± 5.2, 25.2	24.1 ± 3.6, 24.7	0.05

^a^ Mean ± SD, Median.

**TABLE 7:** Microcirculation parameters stratified by macrocirculatory hemodynamics (Pulse pressure).

Microcirculation parameters by pulse pressure (Quartiles)					
Characteristic	Lowest	Low‐Middle	High‐Middle	Highest	*p* ^b^
	(23–45 mmHg)	(46–55 mmHg)	(56–72 mmHg)	(73+ mmHg)	
	*N* = 31^a^	*N* = 24^a^	*N* = 30^a^	*N* = 27^a^	
Age, years	62 ± 14, 65	61 ± 14, 62	60 ± 13, 62	70 ± 8, 72	0.014
MFI	2.88 ± 0.20, 3.00	2.81 ± 0.24, 2.93	2.90 ± 0.15, 3.00	2.84 ± 0.20, 2.92	0.4
MHI	0.11 ± 0.14, 0.00	0.19 ± 0.26, 0.10	0.13 ± 0.19, 0.00	0.18 ± 0.23, 0.12	0.5
PPV	94.1 ± 4.8, 95.2	93.1 ± 6.1, 96.3	95.3 ± 4.4, 96.5	94.2 ± 4.5, 94.2	0.5
PVD	22.5 ± 4.3, 22.3	22.9 ± 4.9, 21.8	23.0 ± 4.5, 21.9	24.2 ± 5.0, 23.9	0.6
TVD	23.9 ± 4.2, 23.1	24.5 ± 4.7, 23.8	24.2 ± 4.9, 22.6	25.7 ± 5.3, 25.7	0.5

^a^ Mean ± SD, Median.

## 4 Discussion

The data presented here provide a core set of sublingual microcirculation measurements using IDF‐HVM in a large cohort of patients with stable CVD. While most HVM studies are in the setting of acute illness [23, 24, 25], our study represents the largest sample of human subjects with stable, chronic CVD [26, 27, 28, 29, 30]. Our findings indicate that sublingual microcirculatory parameters do not vary between age quartiles and most common cardiovascular disease comorbidities, with the exception of MHI, which is lower in diabetic patients. These results are important as they establish baseline values for microcirculatory flow variables measured with a current generation HVM imaging device in healthy patients with chronic CVD.

Our primary finding was that sublingual microcirculatory flow and density were similar across most cardiovascular disease co‐morbidities and age groups. The exception was diabetic patients, who were found to have decreased microcirculatory flow heterogeneity. Our data is in contrast with previous human and experimental studies, which have reported decreases in capillary density within the brain, skeletal muscle, and myocardium with aging and cardiovascular disease [31, 32, 33]. Age related changes in microcirculatory structure and reactivity have been linked to increases in endothelial dysfunction, driven by proinflammatory molecules and reduced nitric oxide bioavailability [16, 34]. Our data is similar to a recently published study of 150 healthy patients, which similarly found no change in sublingual microcirculatory flow with IDF imaging across age groups, except for a slight increase in TVD and decrease in PPD [29]. Notably, our cohort's TVD, PVD, and PPV were 5%–10% lower than those reported by Yang et al. which may be the result of cardiovascular comorbidities as opposed to aging alone. Reynolds et al. also found no difference in sublingual microcirculation parameters relating to age or comorbidity (DM, cirrhosis, CKD) with second‐generation sublingual microcirculation imaging (SDF) [28]. MFI and PPV were similar to our data, but PVD was 3× higher in our study. This likely reflects the increased vessel detection of current‐generation IDF‐HVM technology [35, 36]. Taken together, our results along with the handful of other HVM studies in CVD suggest two potential hypotheses that may warrant further study. Firstly, it is possible that the sublingual microcirculation is not significantly affected by age and/or CVD to the same degree as other organ capillary beds. Second, it may also be possible that while sublingual HVM can detect the profound microcirculatory alterations present in shock, it may not be sensitive enough to detect more subtle changes associated with CVD.

Diabetic patients had decreased sublingual microcirculatory flow heterogeneity (Table 5, Figure 3). Diabetes has well‐described effects on arterioles, capillaries, and the microvasculature, which include hypertrophic remodeling, endothelial dysfunction, glycocalyx alterations, increased capillary permeability, and reduced endothelial nitric oxide bioavailability [37, 38, 39, 40, 41]. These changes result in chronic injury to organ systems and symptomatic pathologies, including renal disease and retinopathy [37]. MHI quantifies the degree to which flow differs between different parts of the same capillary bed, meaning that the decreased MHI observed in diabetic patients may reflect chronic changes in microvascular organization. Some degree of microvascular flow heterogeneity is normal because metabolic activity within organ tissue is inherently heterogenous [42, 43]. In healthy states, the microcirculation dynamically autoregulates to optimize perfusion to meet heterogeneous oxygen demand. This is achieved through mechanisms such as nitric oxide release and subsequent vasodilation [44, 45, 46]. The impaired vasoreactivity observed in diabetes may result in impaired autoregulation, and therefore decreased MHI.

Additionally, several prior studies have identified increased MHI as a mechanism of microvascular dysfunction in acute illness, a phenomenon termed ‘type 1 microcirculatory dysfunction’ [47, 48, 49]. Our study is unique in that it also implicates decreased flow heterogeneity as a relevant mechanism of microcirculatory dysfunction. This finding may reflect the chronic disease state of our study population as opposed to the acute shock population of most other studies. Finally, the effects of diabetes on sublingual microcirculatory density and morphology remain unclear [50, 51, 52, 53, 54]. We did not find a difference in microcirculatory TVD or PVD between diabetic and non‐diabetic subjects. This may reflect the heterogeneity of co‐morbidities in patients with cardiovascular disease and variable severity and impact of chronic conditions.

Observational human studies of sublingual microcirculation in acute illness rarely include baseline microcirculation assessments, making it difficult to determine whether absolute values or changes in microcirculatory parameters are more important. Existing literature has consistently reported lower MFI, PPV, and PVD, along with higher flow heterogeneity in critically ill patients with increased organ injury scores and poor outcomes, but it is unclear how these values compare to those of stable patients with chronic cardiovascular disease [14, 48, 55, 56, 57, 58, 59, 60, 61]. In our cohort, vessel density and flow measurements were 15%–51% higher than those of acutely ill patients with cardiogenic shock [62]. Given our adherence to consensus recommendations for image acquisition and analysis, the large size of our dataset, and the novel focus on chronic cardiovascular disease, we propose that our data represent a baseline reference for sublingual microcirculation in patients most at risk for later development of circulatory shock. This may aid in identifying microcirculatory abnormalities in both the acute and chronic disease settings and could be used in defining a range for future therapeutic targets.

Our study has a few important limitations. We did not include a true control group without CVD, but our data is consistent with recently published literature which primarily studies a healthy patient population [29]. We also recognize that concomitant CVDs could result in more notable microvascular changes, though our present sample size would likely be too small to achieve this more granular sub‐stratification. A larger patient sample would allow for regression analyses that could quantify the impact of individual diseases on microcirculatory blood flow. There are also limitations inherent to HVM. While the sublingual microvascular bed is accessible, it does not perfectly correlate with other organ microvasculature [63].

## 5 Conclusion

Our study defines a baseline reference range of sublingual microcirculation parameters using current generation IDF video microscopy in a large cohort of stable patients with chronic cardiovascular disease. With the exception of diabetes, sublingual parameters did not vary meaningfully between patient subpopulations. Our data also implicates altered microvascular heterogeneity as a mechanism of microvascular dysfunction in diabetic patients. Future studies that evaluate microcirculatory blood flow in patients in acute illness could use this reference range to compare microcirculation parameters in similar patient populations.

## 6 Perspectives

We defined a normal range for sublingual microcirculation parameters in stable patients with chronic cardiovascular disease. With the exception of decreased microvascular heterogeneity in diabetic patients, sublingual microvascular parameters are similar between common CVD cohorts. Future studies may leverage this reference range to study how acute illness states alter microcirculatory density and perfusion relative to the baseline values published here.

## Conflicts of Interest

J.W.W. receives funding from Abiomed Research and serves on Boston Scientific advisory board as well as the Impulse Dynamics speakers bureau.

## Supporting information


**Data S1:** micc70032‐sup‐0001‐Figures.pptx.

**Table S1:** micc70032‐sup‐0001‐TableS1.xlsx.

## Data Availability

The authors have nothing to report.
