# Supplementary material for: Observational Study of Sublingual Microcirculation in Patients With Chronic Cardiovascular Disease
Source: Microcirculation. 2025 Oct 13;32(7):e70032. doi: 10.1111/micc.70032 (PMC12517397; doi:10.1111/micc.70032)
Supplement: Supplementary file 1 — Data S1: micc70032‐sup‐0001‐Figures.pptx. [file MICC-32-e70032-s001.pptx]

## Slide 1
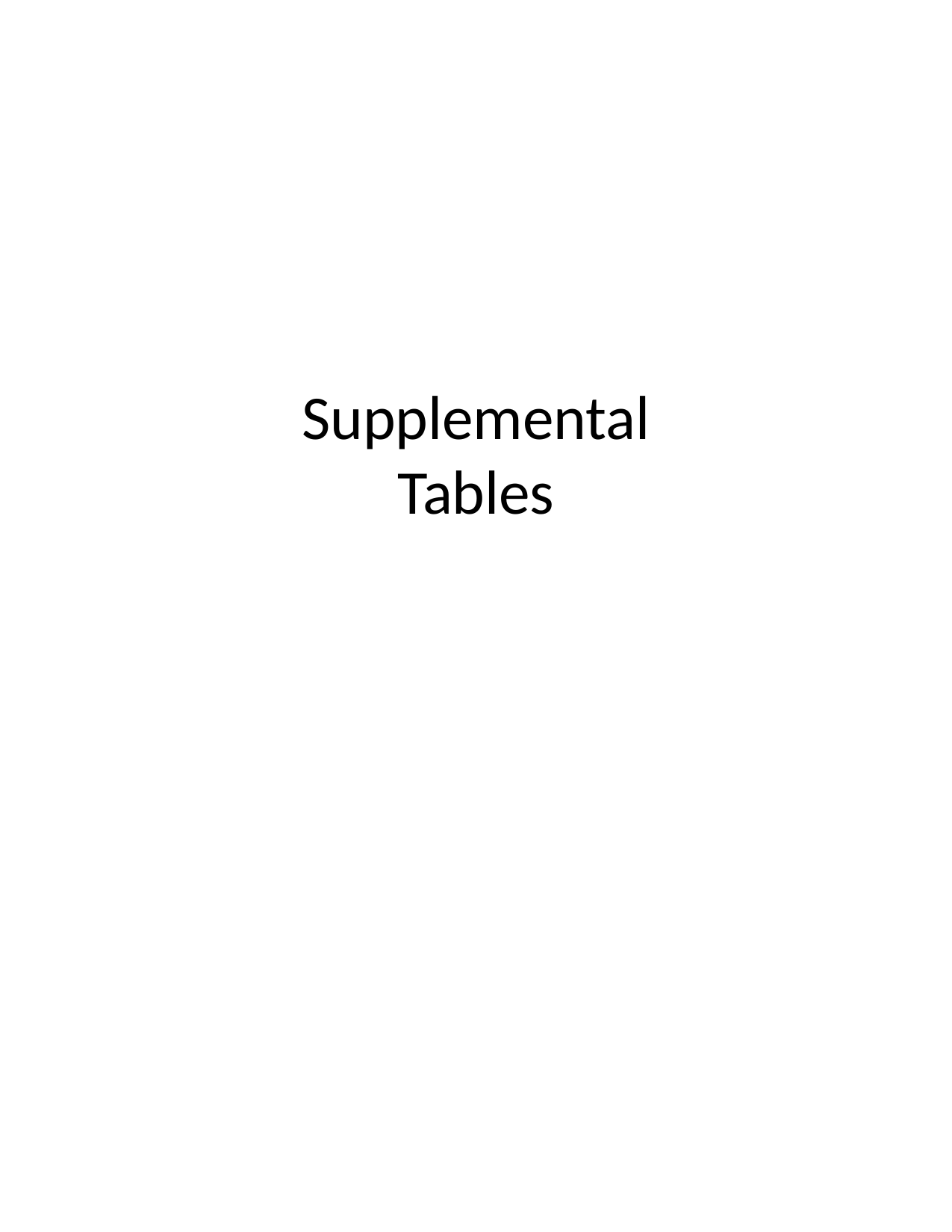

Supplemental Tables

## Slide 2
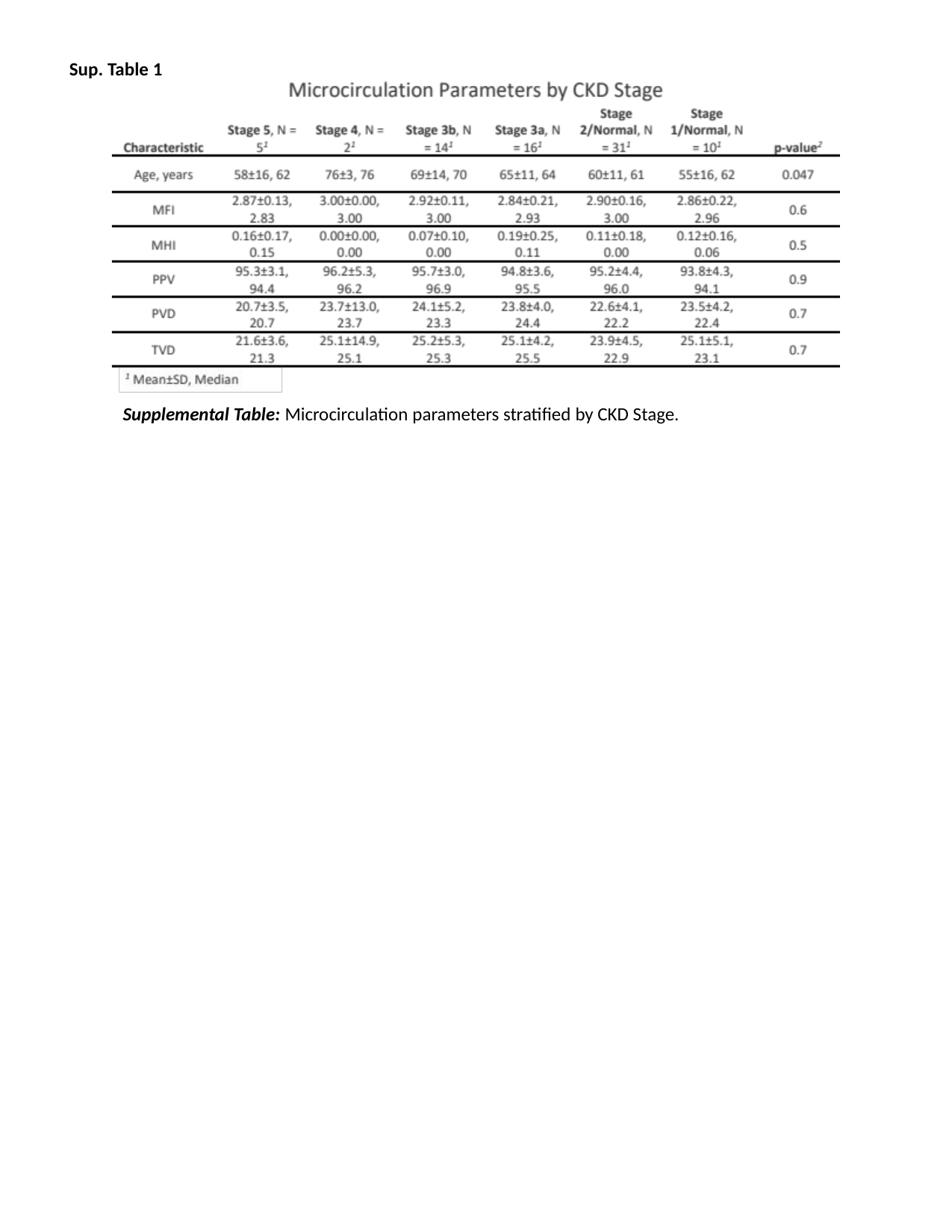

Sup. Table 1
Supplemental Table: Microcirculation parameters stratified by CKD Stage.

## Slide 3
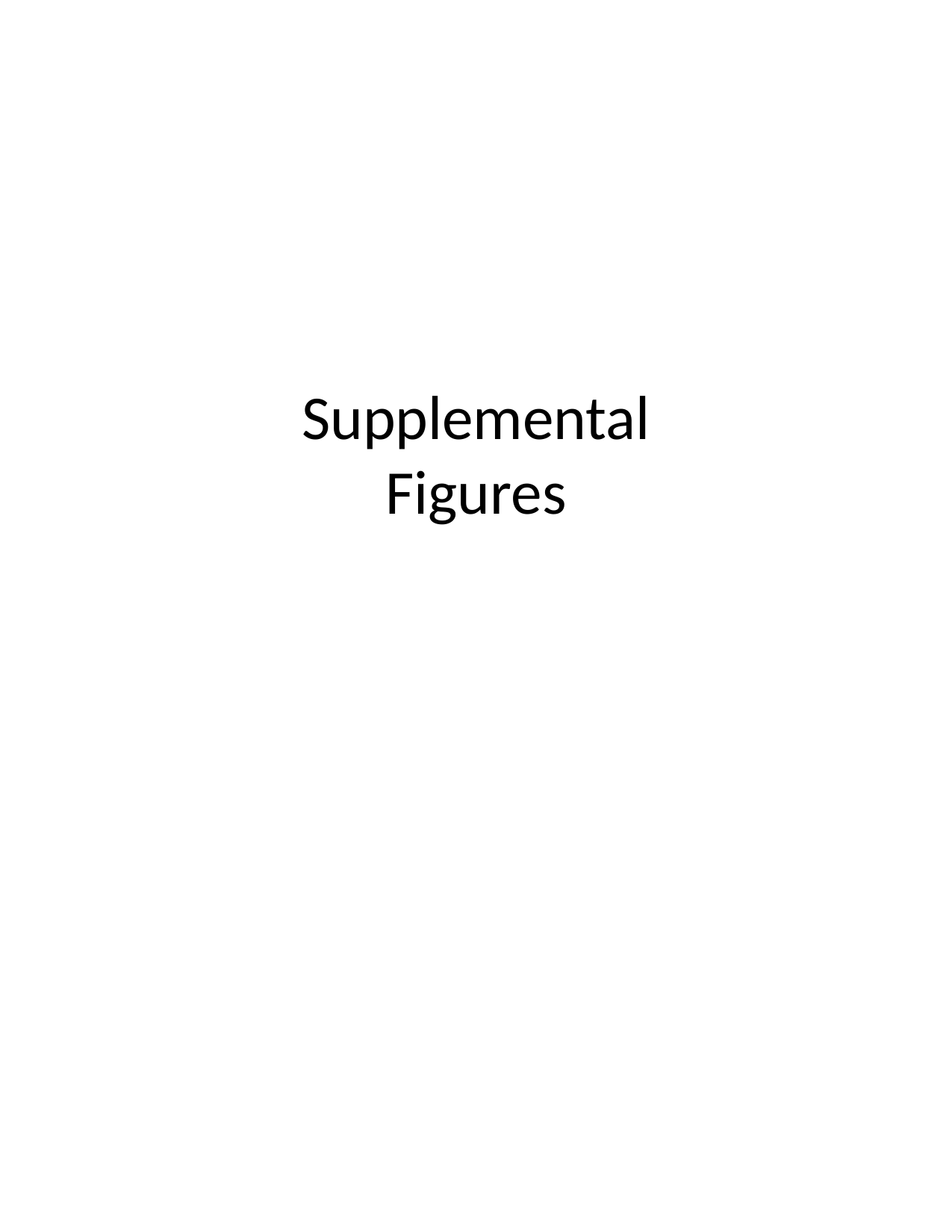

Supplemental Figures

## Slide 4
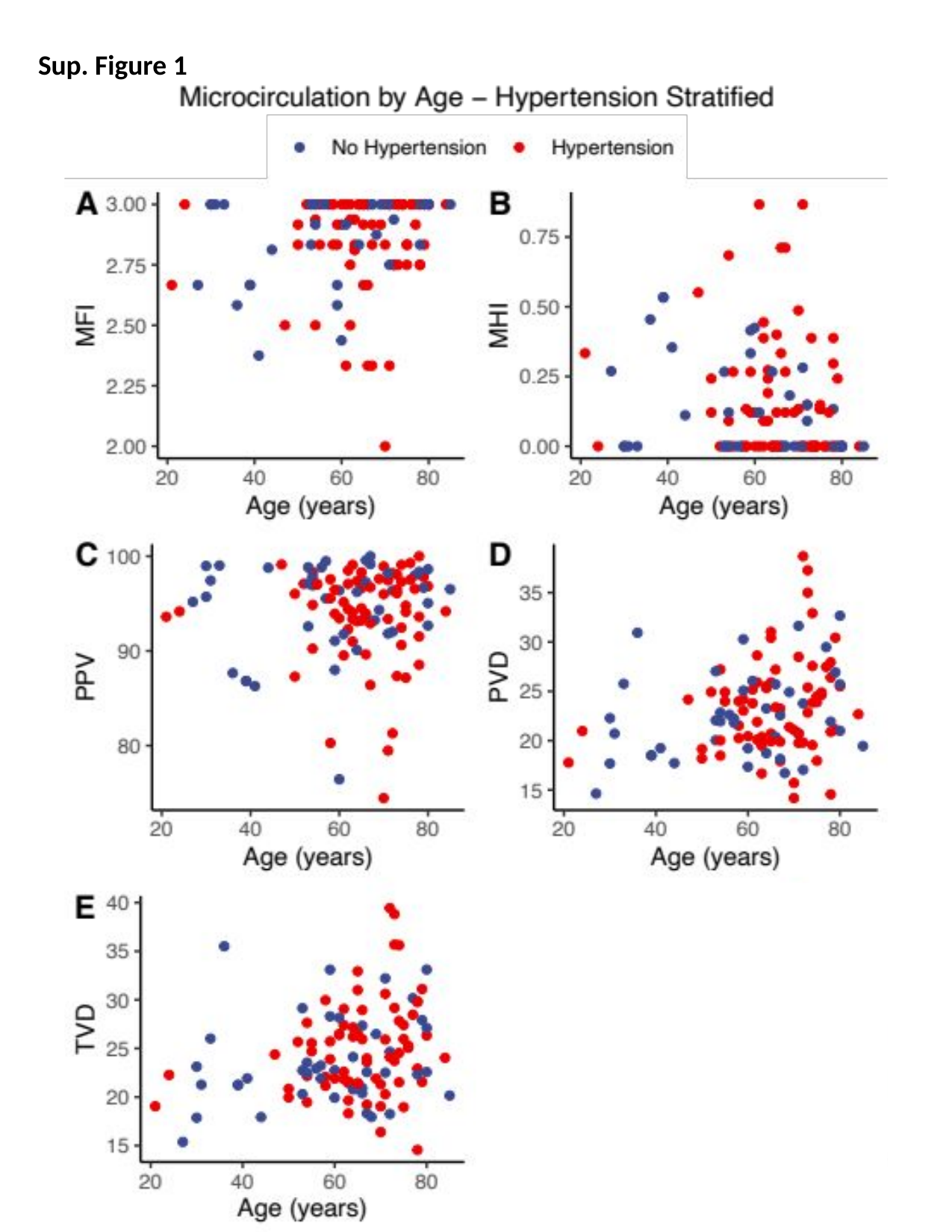

Sup. Figure 1

## Slide 5
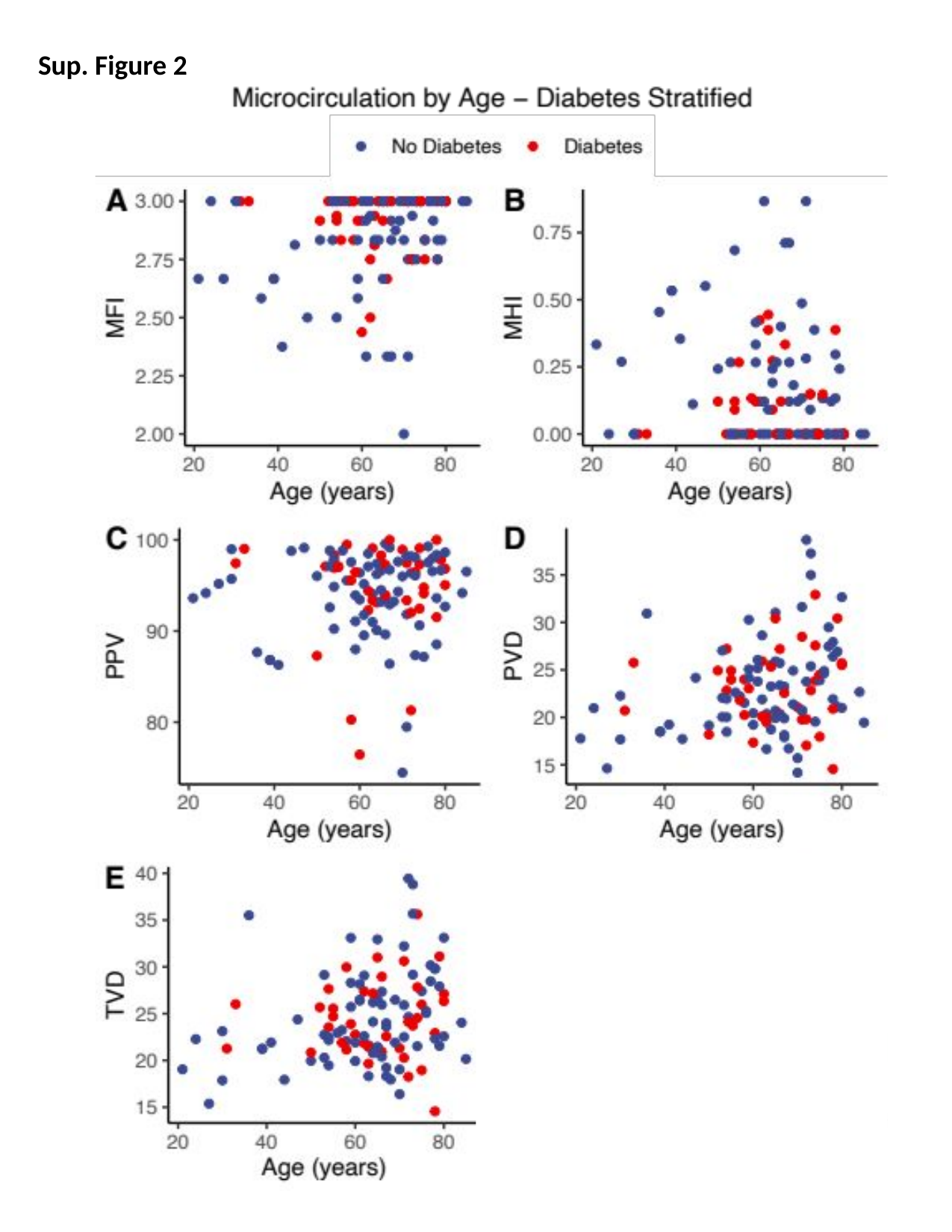

Sup. Figure 2

## Slide 6
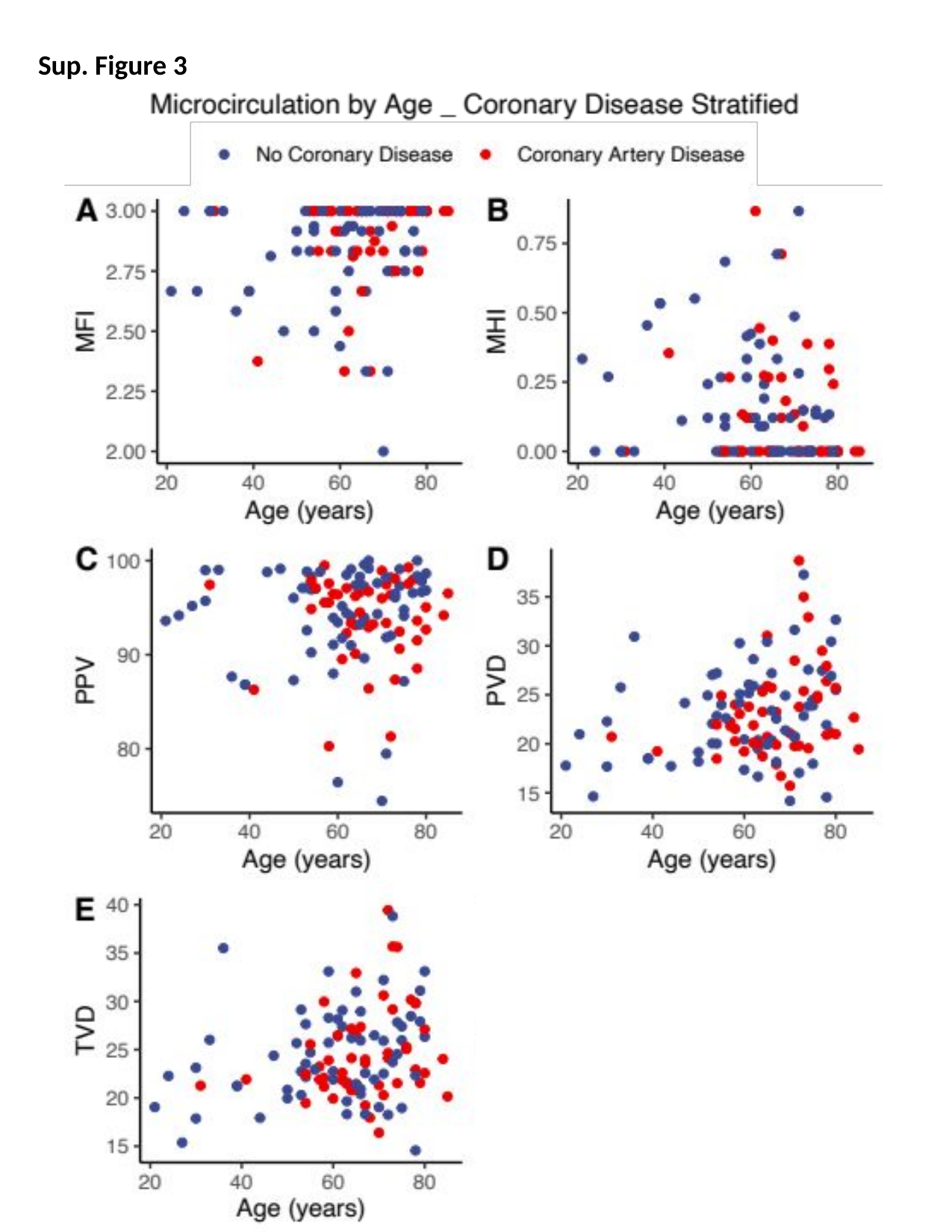

Sup. Figure 3

## Slide 7
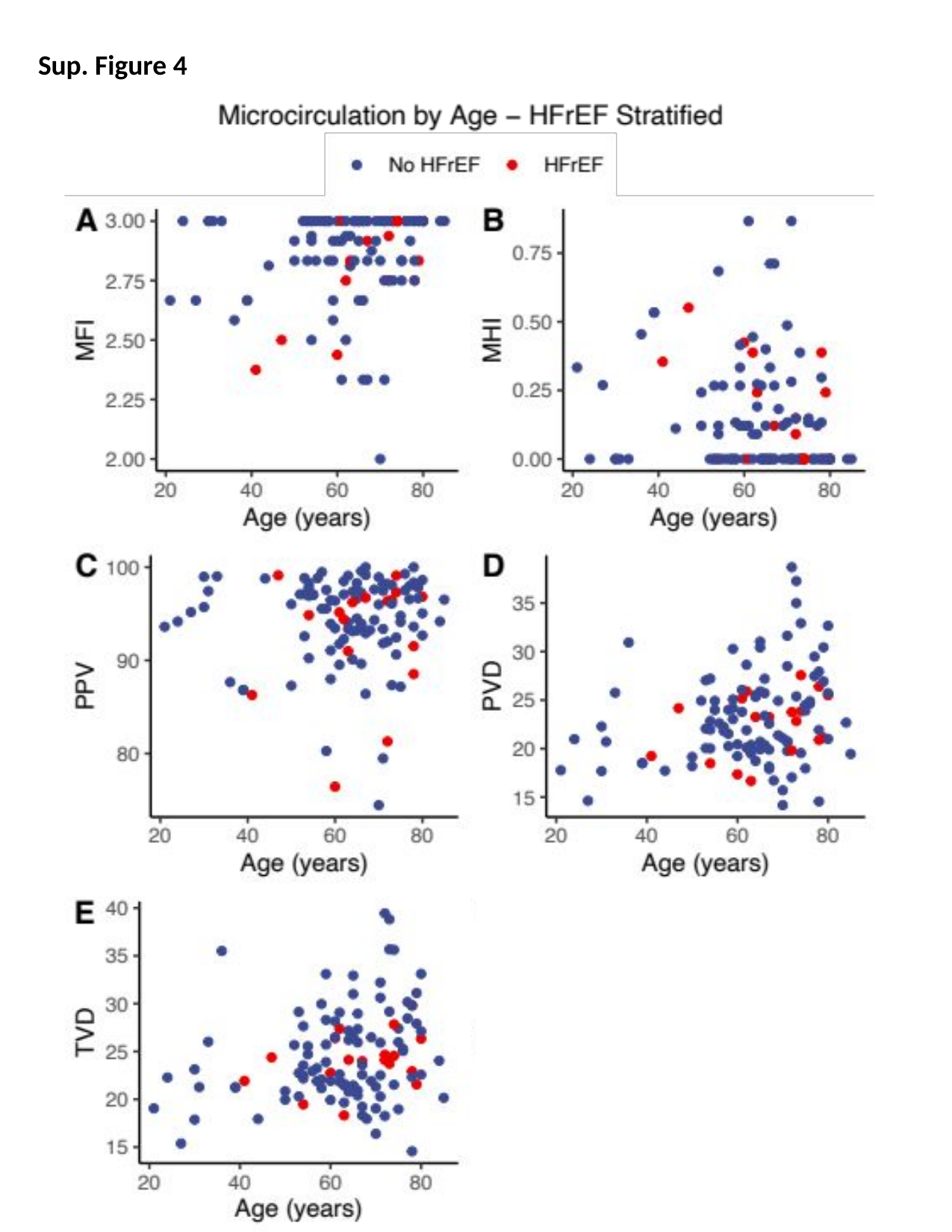

Sup. Figure 4

## Slide 8
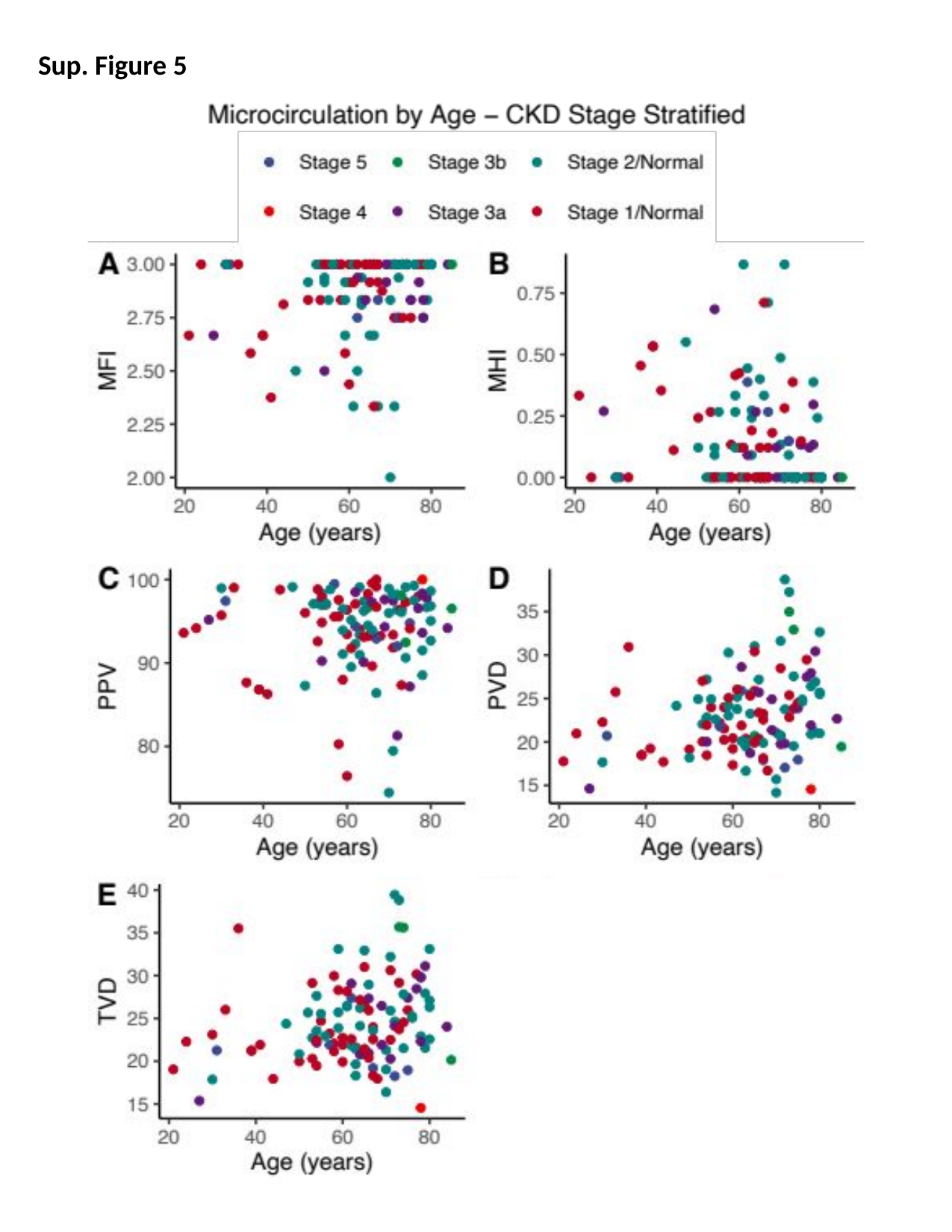

Sup. Figure 5

## Slide 9
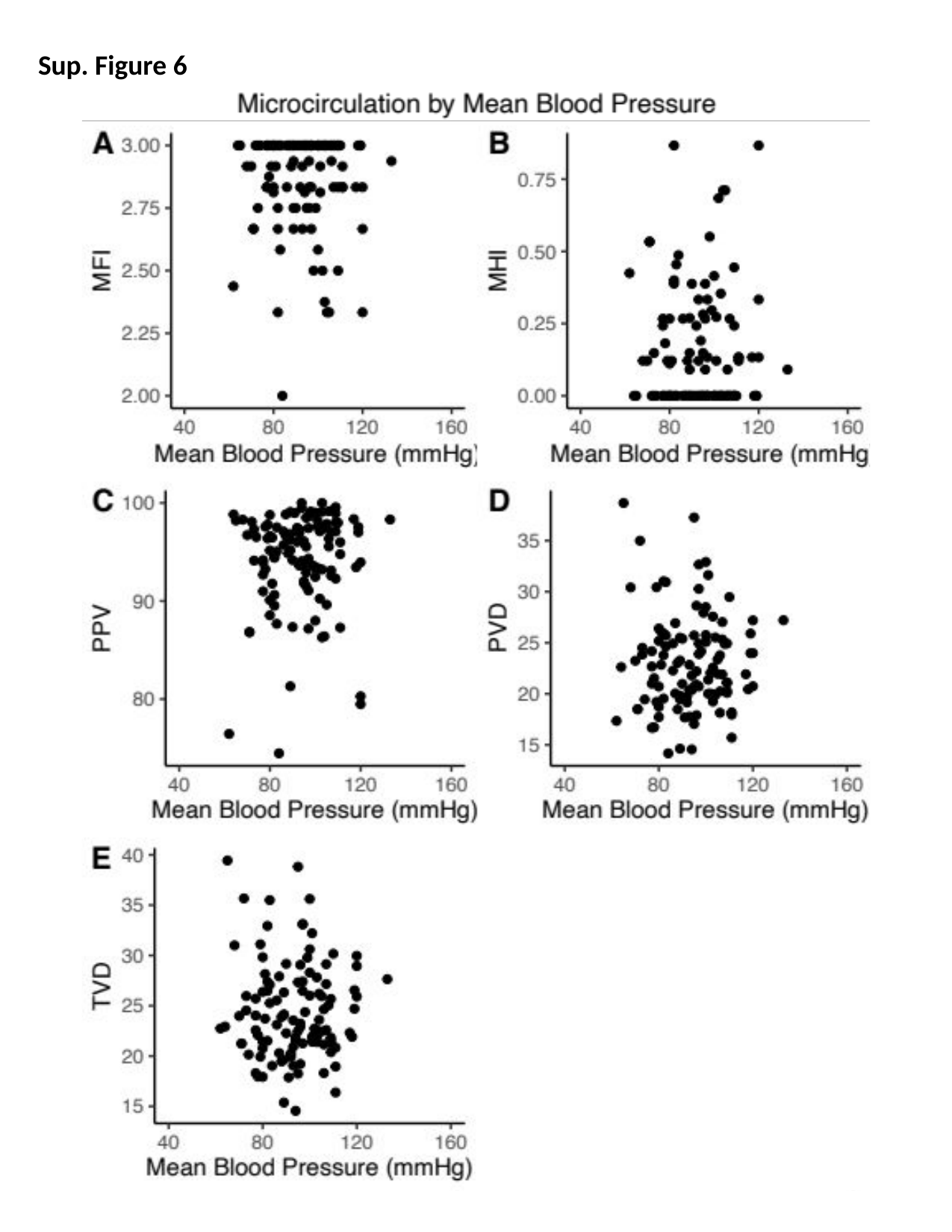

Sup. Figure 6

## Slide 10
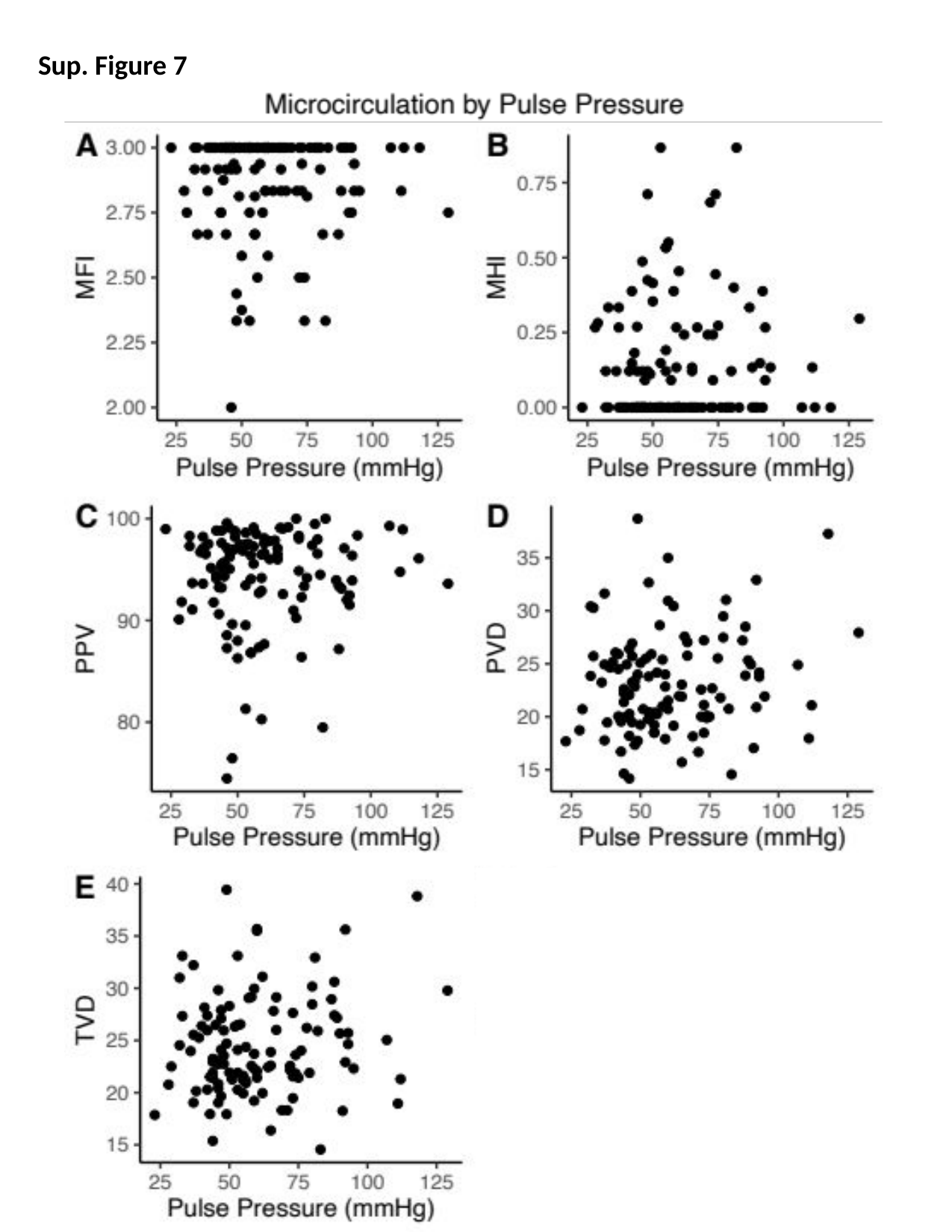

Sup. Figure 7
